# ARe we there yet? Understanding androgen receptor signaling in breast cancer

**DOI:** 10.1038/s41523-020-00190-9

**Published:** 2020-09-25

**Authors:** Anna R. Michmerhuizen, Daniel E. Spratt, Lori J. Pierce, Corey W. Speers

**Affiliations:** 1 Department of Radiation Oncology, University of Michigan, Ann Arbor, MI USA; 2 Cellular and Molecular Biology Program, University of Michigan, Ann Arbor, MI USA; 3 Rogel Cancer Center, University of Michigan, Ann Arbor, MI USA

**Keywords:** Breast cancer, Targeted therapies

## Abstract

The role of androgen receptor (AR) activation and expression is well understood in prostate cancer. In breast cancer, expression and activation of AR is increasingly recognized for its role in cancer development and its importance in promoting cell growth in the presence or absence of estrogen. As both prostate and breast cancers often share a reliance on nuclear hormone signaling, there is increasing appreciation of the overlap between activated cellular pathways in these cancers in response to androgen signaling. Targeting of the androgen receptor as a monotherapy or in combination with other conventional therapies has proven to be an effective clinical strategy for the treatment of patients with prostate cancer, and these therapeutic strategies are increasingly being investigated in breast cancer. This overlap suggests that targeting androgens and AR signaling in other cancer types may also be effective. This manuscript will review the role of AR in various cellular processes that promote tumorigenesis and metastasis, first in prostate cancer and then in breast cancer, as well as discuss ongoing efforts to target AR for the more effective treatment and prevention of cancer, especially breast cancer.

## Introduction

Androgens are expressed at different levels in men and women, and while they are important for proper development, they can also drive tumor growth. Most notably, the role of the androgen receptor (AR) in prostate cancer has been extensively studied. Recent data suggest that AR signaling may also be important in breast cancer, glioblastoma, and additional tumor types with AR expression^1^. In order to develop effective treatment strategies for patients with each of these cancer types, it is important to understand how AR is functioning similarly and differently to drive tumor growth.

AR belongs to the Type I class of nuclear hormone transcription factors along with the estrogen receptor (ER), progesterone receptor (PR), and glucocorticoid receptor (GR)^2^. As is characteristic of this type of receptor, inactive forms of AR are located in the cytoplasm, bound to heat shock proteins (HSPs)^3^. The HSPs are responsible for proper protein folding, prevention of misfolding and maintaining 3D protein structure during events of cellular stress^4,5^. AR, like other receptors in this family, is activated by the binding of androgen molecules to its ligand binding domain (LBD). Androgen binding results in AR homodimerization and translocation into the nucleus, where AR binds to androgen response elements (AREs) resulting in activation and transcription of a variety of downstream genes. Binding of AR results in the activation of diverse signaling pathways, including multiple signaling pathways that have been implicated in cancer, including the PI3K/AKT pathway^6^. AR also contributes to cell growth and proliferation differently in the context or absence of ER, and AR has an influence on cell cycle and DNA damage repair. Further, AR has non-genomic functions that can influence cell growth, migration, metastasis, and apoptosis. Due to its many downstream effects, antiandrogen therapies have long been of therapeutic interest along with combining AR antagonism with conventional chemotherapy or radiation therapy.

## Gene expression and hormone receptor function

The AR has been well characterized as a key driver for the growth of prostate cancer in men. In this context, castration or androgen deprivation therapy (ADT) is a first line of therapy for men with metastatic prostate cancer. Despite the efficacy of ADT, resistance is near-universal. In some men, resistance can be mediated by AR amplification^7^, and others develop mutations in the LBD of AR in response to antiandrogen treatment^8^. These mutations can render cells refractory to androgen deprivation as there is constitutive AR activation, even in the absence of androgens. This results in activation of AR including AR binding to AREs and constitutive AR-regulated gene expression. More recently, a role for AR in the progression of breast cancer has been described. While AR’s function has not been fully characterized in breast cancer, work done in prostate cancer informs the potential function of AR in breast cancer.

Similar to the role that AR plays in prostate cancer development and progression, the ER has been recognized for the integral role that it plays in driving the development of the majority of breast cancers^9^. Breast cancers that express the ER (ER+) grow in response to the presence of estrogen and are more responsive to endocrine ablation^10,11^. This understanding led to some of the first “molecularly targeted therapies” that established the use of aromatase inhibitors (AIs) or selective estrogen receptor modulators (SERMs), which block the production and signaling of estrogen^12^. AIs and SERMs have been used as effective therapies for women with tumors that express ER^13,14^. Despite having identified the presence of AR expression in breast cancer many years ago^15^, little is known about the role of androgen signaling in breast cancer, though its importance as a potentially effective therapeutic target is increasingly appreciated and will be discussed herein. We begin with a review of the various processes known to be mediated by AR signaling, as recent studies have shed light on the role of AR with other pathways known to be abrogated in cancer.

## Transcription factor and protein interactions

### AR and FOXA1

#### Prostate cancer

FOXA1 is a transcription factor which plays an important role in aiding the binding of hormone receptors to their target DNA^16^. More recently, three distinct classes of alterations in FOXA1 have been described in prostate cancer, each with unique structural and phenotypic consequences^17^. The Class-1 activating mutations originate in early prostate cancer without alterations in ETS or SPOP and are found in the wing-2 region of the DNA-binding forkhead domain. Functionally these mutations allow for enhanced chromatin mobility and binding frequency and strongly transactivate a luminal AR program. The second class of activating mutations are found in metastatic prostate cancer and are characterized by a truncated C-terminal domain. These mutations increase FOXA1 DNA affinity and promote metastasis by activating the Wnt pathway through TLE3 inactivation. The final class of FOXA1 genomic rearrangements are characterized by duplications and translocations within the FOXA1 locus that reconfigure regulatory elements (FOXA1 mastermind elements) to drive overexpression of FOXA1. This third class of alterations is found primarily in metastatic prostate cancer and further underscores the interaction and significance of AR and FOXA1 protein interactions^17^. Similar classes of alterations also been observed in breast cancer^1^. In prostate cancer, FOXA1 also influences the ability of AR to bind DNA and control cell cycle progression. FOXA1 binds to genes necessary for growth of castration-resistant prostate cancer (CRPC), suggesting that FOXA1 is responsible for driving cell cycle progression in CRPC both from G1 to S and G2 to M^18^. FOXA1 also facilitates cell cycle progression from G2 to M by acting as a cofactor for AR^18^. Unsurprisingly, there is also significant overlap between genomic binding sites occupied by AR and FOXA1^19^. While AR binds to many DNA regions independent of FOXA1, DNA-binding sites often require the presence of FOXA1 for AR recruitment^19^. Therefore, loss of FOXA1 results in the inability of AR to bind many DNA loci^19^. Using H3K4me2 ChIP analyses, Sahu et al. found that there were H3K4me2 marks at ~70% of sites shared by AR and FOXA1^19^. Furthermore, staining of FOXA1 has been shown to correlate with disease outcomes in prostate cancer patients, where even with high AR staining, low FOXA1 is associated with good prognoses, and strong FOXA1 staining correlates with poor prognoses^19^, indicating that FOXA1 may have an important effect on AR signaling and tumor progression. Levels of FOXA1 are also elevated in prostate tumors and metastases, and overexpression of FOXA1 in prostate cancer cell lines results in increased AR binding at novel sites that have high chromatin accessibility^20^. These results suggest that increased levels of FOXA1 enhance AR binding to novel sites in order to facilitate cancer cell growth^20^ and implicate the importance of FOXA1 on AR function and tumor progression.

#### Breast cancer

FOXA1 is also essential for the growth of ER+ breast cancer cell lines^21^. Similar to prostate cancer, ChIP-seq studies have shown that there is extensive overlap between locations of AR and FOXA1 binding in breast cancer cells^22^. The function of AR in breast cancer is also dependent upon FOXA1, as silencing of FOXA1 inhibits AR binding of target DNA as well as cell growth^22^. In addition, FOXA1 functions as a transcription factor, playing an important role in aiding binding of hormone receptors, including ER and AR, to their target DNA^16,23^. When expressed with AR, FOXA1 may direct AR binding at sites of ER binding in luminal tumors^24^. Notably, co-expression of AR and FOXA1 was observed by immunohistochemistry (IHC) in ~15% of triple-negative breast cancer (TNBC) patients^24^, and AR-positive (AR+), FOXA1-positive (FOXA1+) patients had a significant decrease in recurrence-free survival and overall survival compared to TNBC patients^25^. These findings suggest that when co-expressed in TNBC, AR, and FOXA1 may be mediating an estrogen-like gene signature similar to those expressed in luminal breast cancers. FOXA1 has been studied extensively in the context of ER chromatin binding, and ER binding is dependent on FOXA1 in the presence or absence of ligand^23^. Further, similar to findings in prostate cancer, 1.8% of breast cancers harbor mutations in FOXA1, and amplifications of the FOXA1 gene locus have been observed in breast and prostate cancers^26^. Notably most identified mutations are in the forkhead domain of FOXA1, and tumors in this study were exclusively ER+. The implications of these mutations, however, is still under investigation in breast cancer. Interestingly, differences exist between the function of FOXA1 in directing AR binding in breast versus prostate cancers, and future studies may investigate the varied roles of FOXA1 in directing AR binding in TNBC and prostate cancer, in addition to investigating the role of AR when co-expressed with ER. Current literature suggests, however, that regardless of tumor type, FOXA1 is an important cofactor for directing the transcriptional activity of AR.

### AR and PTEN

#### Prostate cancer

Expression of AR with PTEN has also been investigated in prostate cancer. In prostate cancer patients, high AR expression with low PTEN expression is associated with poor clinical outcomes^27^. In prostate tumors, with loss of *PTEN*, there are decreased levels of AR signaling^28^. Inhibition of PI3K in these tumors results in increased levels of AR signaling through loss of human epidermal growth factor receptor 2 (HER2)-mediated feedback inhibition of AR^28^. A direct physical interaction between AR and PTEN in low passage LNCaP cells has been shown to inhibit nuclear translocation of AR resulting in an increase in degradation of AR protein^29^. A pilot study suggested that high expression of both AR and PTEN in patients with advanced prostate cancer was associated with a higher risk of relapse at 30 months after surgery (85.7% of high AR and PTEN expressing patients verses 16.6% in patients with low AR and PTEN expression)^30^. Further, combination therapy with both antiandrogen (bicalutamide) and PTEN induction was shown to reduce prostate-specific antigen (PSA) promoter activity compared to PTEN alone^31^. Sequencing of metastatic-CRPC (mCRPC) patients revealed that *AR* and *PTEN* are among the most commonly aberrant genes, along with the ETS family and *TP53*^32^. Therefore, these data suggest that both AR and PTEN may influence prostate tumor growth and progression.

#### Breast cancer

There are opposing findings when comparing AR and PTEN transcript expression in prostate verses breast cancer. In breast cancer, there is an AR-binding motif located in the *PTEN* promoter, and there is a positive correlation between AR and PTEN transcript levels^27^. In addition, high expression of AR and PTEN is correlated with better clinical outcomes for breast cancer patients^27^. Interestingly, in AR + TNBC, AR interacts at an ARE located in the promoter of ERβ^33^, and ERβ also plays a role in regulation of PTEN expression to control tumor growth^34^. The interaction between AR and PTEN may be context specific and important for predicting outcomes for patients with AR+ disease: where AR expression is associated with disease progression in prostate cancer^7^, PTEN loss is also correlated with poor outcomes^27,35,36^. In breast cancer, however, loss of PTEN is also correlated with negative ER and PR status, and PTEN loss is associated with breast tumor progression^37^. Therefore, these results suggest that the function of PTEN may be context specific and understanding the nuances in situational signaling of AR may help elucidate the role for PTEN in AR+ disease progression.

### Non-genomic AR functions

#### Prostate cancer

Prostate cancer cells exhibit rapid proliferation responses in response to androgen stimulation, suggesting non-genomic AR signaling. Upon activation with androgens or estrogens, cytoplasmic AR can activate MAPK/ERK signaling through an association with Src^38^. The activation of the Src/ERK pathway is dependent on androgen concentration (0.1–10 nM) and is inhibited at high concentrations (100 nM)^39^. Treatment with dihydrotestosterone (DHT) also induces rapid ERK1/2 phosphorylation; however, MAPK activation can be blocked pharmacologically using a MEK inhibitor, suggesting AR is activating the Raf1-MEK pathway resulting in MAPK activation^40^. Further, AR can also activate the phosphatidyl-inositol 3-kinase (PI3K)/Akt pathway leading to activation of mammalian target of rapamycin (mTOR)^41^. In addition, androgens can interact at the plasma membrane which is associated with the modulation of intracellular calcium and cAMP levels^41,42^. Many membrane-bound G-protein coupled receptors are also responsive to androgen treatment, leading to an increase in apoptosis^43^, phosphorylation of ERK^44^, or reduced cell migration and metastasis^45^. Together, these findings suggest that AR may also function within the cytoplasm or at the membrane to activate non-genomic functions.

#### Breast cancer

Similar to non-genomic AR functions in prostate cancer, the cytoplasmic roles of AR have also been investigated in breast cancer. Chia et al. demonstrated that AR is necessary and sufficient for ERK phosphorylation following DHT stimulation in MDA-MB-453 and HCC-1954 cells^46^. Further, inhibition of AR resulted in decreased levels of phospho-Elk1, phospho-RSK, and c-FOS in xenograft tumors and in patient tumors, corresponding to a decrease in ERK target proteins^46^. In TNBC, AR inhibition has also been shown to modulate the activity of the Ca^2+^-activated K^+^ channel, K_Ca_1.1, which is associated with breast cancer invasion and metastasis^47,48^. Multiple groups have also studied the role of cytoplasmic AR phosphorylation^49,50^; however, additional work is required to understand how AR modifications influence cellular function and localization. At the membrane, many receptors mediate rapid responses to androgen signaling, representing novel membrane-ARs^51,52^. These signals, however, are complex as agonistic verses antagonistic effects are dependent on receptor stoichiometry^52^. Furthermore, AR is expressed in fibrosarcoma cells; however, a significant portion of AR is transcriptionally incompetent and does not bind to AREs upon activation. Rather, there is crosstalk between EGFR and AR, and treatment with bicalutamide decreases xenograft tumor growth^53^. Together these data from multiple cancer models suggest that AR has non-genomic functions affecting tumor growth both in prostate and breast cancer which warrant further investigation.

## Cell growth and proliferation

### Androgens and AR splice variants

#### Prostate cancer

A number of AR splice variants have been identified, and they play an important role in the development of CRPC. The gene encoding AR is located on the X chromosome, encoding nine exons that produce the full-length AR transcript^54^. Aberrant splicing of AR pre-mRNA, however, can result in the production of AR isoforms that are constitutively active. These isoforms can drive an AR transcriptional program even in the absence of androgen signaling, resulting in androgen independent tumor growth^55,56^. AR variants (ARVs) are present both in prostate cancer and breast cancer, and these variants commonly are truncated or have mutations in the AR LBD^57^. In addition, AR transcripts can have aberrant splicing, resulting in skipped exons^58^. ChIP studies have demonstrated that splice variants, including AR-V7, are able to bind canonical AREs as well as unique regions of additional genes^58^. AR splice variants have been shown to require expression of full-length AR (AR-FL) suggesting that a balance between ARV and AR-FL expression is required for resistance in prostate cancer models^59^.

The most common splice variant, AR-V7, lacks an LBD^60^. Clinically, in a cohort of prostate cancer patients treated with enzalutamide or abiraterone acetate, 39 patients (19%) had detectable levels of AR-V7 in circulating tumor cells (CTC)^61^. Patients with AR-V7 expression had lower PSA response rates and worse survival compared to AR-V7 negative patients^61^. In addition to reliance on AR-splice variants, resistance to ADT is also mediated through signaling of additional hormone receptors. The GR has been shown to be increasingly present in androgen-deprived prostate cancer patients (78% vs. 38% of untreated patients)^62^, and expression of GR is increased in xenografts that are resistant to ARN-509 (apalutamide)^63^. In addition, there is overlap between AR and GR binding at classic response elements as well as regulation by both DHT and dexamethasone, a GR agonist^63^. In prostate cancer cells, stimulation with dexamethasone in the presence of enzalutamide resulted in expression of AR target genes^63^, providing further evidence that GR signaling could compensate for AR in the presence of AR-antagonists.

#### Breast cancer

There are significantly fewer AR mutations observed in TNBC compared to CRPC; however, AR splice variants are still common. In breast cancer, AR-V7 is most highly expressed splice variant in basal tumors compared to other tumor types, with the lowest expression in luminal tumors^60^. Little is known about how AR-V7 may be contributing to antiandrogen resistance in AR+ TNBC or if it is functioning similarly to its observed role in CRPC^64^. In HER2-enriched patients, however, high AR-V7 expression is associated with significantly higher metastasis-free survival and disease-specific survival^60^. Therefore, the ability of a tumor to produce its own androgens, as well as its reliance on splice variants may also play an important role in understanding how AR is functioning to drive tumor growth in the context of ADT or antiandrogen therapies.

Importantly, differences also exist in preclinical cell lines used to study AR+ breast cancers. While common cell lines, including MDA-MB-453, MDA-MB-231, ZR-75-1, MFM-223, MCF-7, and T47D, have varying levels of AR-FL expression, ARV expression also varies widely among cell lines—both in total ARV expression and expression of specific ARVs^65^. Notably, MDA-MB-453 cells contain the AR-Q865H variant which harbors a mutation in the AR LBD^66^, demonstrating the importance of considering the influence of ARV expression in laboratory studies. Furthermore, understanding the similarities and differences of how ARVs may be influencing AR expression and contributing to breast tumorigenesis will be important.

### Estrogen influence on androgen signaling in breast cancer

#### AR+, ER+ cancers

Breast tumors that are ER+ are more likely to be AR+ compared to tumors that are ER−^67^, and AR status is related to ER and PR status but independent of the status of HER2^68^. Interestingly, patients with AR:ER ratios ≥2 have worse disease-free survival compared to patients with lower AR:ER ratios in the presence of antiestrogen therapies or chemotherapy treatment^69^. Defining expression of AR and ER, however, is challenging, and results vary widely depending on the assays (including IHC, radioimmunoassay, and reverse-phase protein array^67^) and cut-offs used to define positivity. Clinically, ER expression is measured by IHC, and ER+ tumors are defined as those with >1% of tumor cells with positive nuclei^70^. AR positivity, however, has been defined with varying cut-off levels from 1 to 75%^67,71^. The prognostic role of AR in breast cancer remains unclear. A recent study has demonstrated that >78% AR positivity is required to accurately assess the prognostic role of AR in ER+ cancers, with ER+ patients that have ≥78% AR positivity having the best survival outcomes^72^. In other studies, however, breast cancer patients with AR+ tumors have better overall survival at both 3 and 5 years compared to patients with AR− tumors, regardless of ER expression^67^. These data suggest that the role of AR in driving breast cancer growth may differ in the presence or absence of ER and that antagonizing AR may have different effects depending on the level of AR expression.

AR expression in ER+ breast cancers antagonizes the signaling of mitogenic ERα, and AR expression leads to the upregulation of ERβ^33^. In ER+ breast cancer, AR binds at an ARE located in the promoter of the ERβ gene, resulting in increased ERβ expression^33^. Interestingly, the presence of ERβ has been shown to inhibit transcriptional activity of ERα^73^, therefore, suggesting that AR-regulated increased activity of ERβ may indirectly influence ERα activity. Similarly, in prostate tissue, ERβ is thought to play an antagonistic role to AR, resulting in the suppression of cellular proliferation and the promotion of apoptosis^74^. ERβ is also important for the control of cell cycle progression and arrest^75,76^, indicating that increasing ERβ expression may be a therapeutic strategy in prostate cancer^77^.

In contrast to early studies suggesting high AR expression is associated with improved outcomes, recent data suggest high AR expression may be associated with therapy resistance, including endocrine therapy resistance. Indeed, De Amicis et al. first reported the positive correlation between high AR expression and tamoxifen resistance, suggesting that tumors with a high AR:ER ratio are more likely to be resistant to antiestrogen therapies, which are common first line of therapy for ER+ tumors^78^. Patients resistant to tamoxifen with AR:ERα ratios ≥2 have worse disease-free survival, and disease-specific survival^79^. Interestingly, in tamoxifen-resistant MCF-7 cells, loss of AR signaling by AR knockdown, but not treatment with enzalutamide, restored sensitivity to tamoxifen^80^. These results suggest that AR expression may be a mechanism of hormone therapy resistance, and therefore a therapeutic target in resistant hormone receptor positive breast cancers.

Anti-AR therapy is of increasing clinical interest. AR inhibition may be an effective strategy for growth inhibition of AR+, ER+ breast tumors. AR inhibition with enzalutamide has been shown to be synergistic with tamoxifen- or fulvestrant-mediated ER inhibition, in addition to controlling growth of tamoxifen-resistant MCF-7 cells in vitro and in vivo in an AR+, ER+ patient-derived xenograft model^81^. Enzalutamide has been shown to be effective in AR+ breast tumors, including ER+ (MCF-7) cells and ER− (MDA-MB-453) cells^82^. ChIP analyses demonstrate that there is extensive overlap between AR and ER binding sites after E2 stimulation in MCF-7 cells^81^. Interestingly, however, AR binding was different based on stimulation with DHT or E2 in MCF-7 cells suggesting that AR may regulate a unique transcriptional program in the absence of estrogen signaling, providing additional evidence for synergism between antiestrogen and antiandrogen therapies^81^. These results indicate that targeting AR in combination with anti-ER therapies may be an effective therapeutic strategy for patients with AR+, ER+ breast cancers.

Functionally, AR and ER share many similarities in their signaling pathways, including the mechanism of receptor activation, as both receptors are activated through ligand binding^83^. ER and AR recognize similar sequences of DNA: where ER binds to 5'-AGGTCA-3', AR recognizes the 5'-AGAACA-3' sequence^83,84^. Further, in breast cancer, both AR and ER require similar cofactors for the activation of common signaling pathways^83^. Binding of AR or ER can activate MAPK signaling, among other pathways^83^, and due to their similar structure and signaling function, both hormone receptors are in competition within the cell for the binding of scaffold proteins and cofactors^83^. While AR and ER share many similarities, there may be important differences determining their role in driving tumor growth.

#### AR+, ER− cancer

The function of AR in breast cancer appears to be dependent upon its co-expression with ER, as there is evidence for varying effects of AR on the growth of breast cancer cells in the presence or absence of ER. Indeed, while AR is co-expressed with ER in 70–90% of breast tumors, AR is only expressed in 15–30% of ER-negative breast tumors^85^. Breast cancers that do not express ER, PR, and HER2 have been traditionally described as TNBC. Recently, however, a subtype of TNBC has been established which is characterized by luminal AR expression^86,87^. In studies with AR+ human breast cancer cell lines, androgens had both proliferative and antiproliferative effects depending on the cell line of interest^88^. More recently, however, multiple groups have demonstrated that targeting AR in AR+ TNBC is an effective treatment strategy both in vitro and in vivo^82,89,90^. Interestingly, in AR+ TNBC, ~30% of patients have expression of ERβ^91^, and ERβ expression has been shown to increase the efficacy of antiandrogens in AR+ TNBC cells^92^. Together these data demonstrate the importance of AR in driving growth of AR+ TNBCs.

While AR expression has been increasingly recognized in AR+, ER− breast cancers, the specific role of AR signaling is not well understood. Some studies suggest an important role for AR in signaling in the absence of ER^93^. In an analysis of AR+, ER− MDA-MB-453 cells, the AR cistrome was found to be more similar to that of ER in MCF-7 (AR−/ER+) cells compared to the AR cistrome in LNCaP prostate cancer cells^22^. Therefore, AR may function in place of ER in AR+, ER− breast cancer^22^, having a distinct role in AR+ TNBC compared to prostate cancer. AR may also be important for promoting the cancer stem cell-like (CSC-like) population in TNBC, in addition to reducing the levels of detachment-induced apoptosis in cells grown in forced suspension compared to attachment conditions^94^. These results suggest that AR blockade may be effective in combination with paclitaxel to target CSC-like cells and reduce tumor recurrence compared to paclitaxel treatment alone^94^. In addition, AR is commonly enriched in breast cancers overexpressing HER2, indicating a role for AR in activation of HER2 and Wnt signaling^89,95^. Therefore, AR expression may be an important target for directing treatments for patients with ER- breast cancer.

## DNA damage repair

### Prostate cancer

While the mechanism of AR in response to DNA damage is just beginning to be uncovered in breast cancer, the mechanistic role of AR in DNA damage repair has been more extensively characterized in prostate cancer. Goodwin et al. found that AR is activated in response to reactive oxygen species (ROS) and DNA damage^96^. Additionally, in response to ionizing radiation, CRPC cells have enhanced DNA repair and decreased DNA damage^97^. AR activation results in the expression of DNA damage repair genes including *PRKDC*, encoding DNA-dependent protein kinase catalytic subunit (DNA-PKcs), an essential protein necessary for nonhomologous end joining (NHEJ) repair of double-stranded DNA (dsDNA) breaks^96^. In addition, treatment with radiation and androgens results in the upregulation of *XRCC2* and *XRCC3*, two genes important for homologous recombination (HR)^96^. Conversely, antiandrogen treatment results in decreased DNA repair in cells and increased levels of dsDNA breaks^97^. The same group also showed that treatment with AR inhibitors results in increased radiosensitivity and decreased NHEJ-mediated recombination suggesting that AR is involved in NHEJ-mediated repair of dsDNA breaks^97^. DNA-PKcs has been shown to function in complex with Ku70 and Ku80 to respond to DNA damage. Interestingly, DNA-PKcs physically interacts with AR; however, this interaction does not require the presence of DNA^98^. This suggests that AR regulation of the DNA damage response may not be completely dependent on AR-mediated transcriptional regulation of DNA damage response genes. Following androgen stimulation in prostate cancer cells, AR is recruited to enhancer elements, along with DNA-PKcs, coregulator p300, and RNA Pol II suggesting that the interaction of AR and DNA-PKcs may be important for the regulation of specific transcriptional programming^98^. Therefore, an interaction between AR and DNA-PKcs may also be important for AR’s role in the repair of DNA damage. In patient tissue, castration resulted in the downregulation of Ku70 protein levels, impairing NHEJ^99^. AR regulates Ku70 levels in prostate tissue, and due to the critical role of Ku70 in effective NHEJ, downregulation of this protein abrogates NHEJ-mediated repair^99^. Collectively these data suggest that AR signaling plays an important role in the repair of dsDNA breaks, at least in part through interactions with Ku70/Ku80 and DNA-PKcs, members of the NHEJ repair pathway.

### Breast cancer

Recent data in breast cancer suggest that loss of AR signaling through knockdown or pharmacologic inhibition with enzalutamide or seviteronel results in increased sensitivity to ionizing radiation^100,101^. In addition, AR mRNA levels correlate with survival following radiation treatment, and AR is important for regulating the DNA damage response in AR+ breast cancer cell lines^102^. Pharmacologic AR inhibition results in delayed repair of dsDNA breaks following ionizing radiation, suggesting that AR is influencing dsDNA damage repair. Additionally, AR inhibition with enzalutamide decreases levels of phosphorylated DNA-PKcs following radiation, indicating that NHEJ may be important for the repair of radiation-induced dsDNA breaks in breast cancer^100^. Although some similarities exist between the role of AR in DNA damage repair in prostate and breast cancers, a full characterization of the similarities and differences is still ongoing.

## Cell cycle regulation

### Prostate cancer

Cell cycle progression is driven by the rising and falling in levels of cyclins and cyclin dependent kinase (CDKs), in addition to the activation of these proteins^103^. In prostate cancer, AR is regulated in a cell-cycle-dependent manner^104^. Nuclear transactivation of AR is highest in G1 and decreases in S phase, while the same changes occur in AR phosphorylation and cellular localization^104^. Further, CDK1 has been shown to phosphorylate AR on S308 in response to ligand binding^104^. The phosphorylation results in changes in AR chromatin localization^104^. AR signaling is responsible for the activation of genes controlling the G1–S transition^105^. Specifically, AR is responsible for G1 CDK activation and the phosphorylation of retinoblastoma (pRb), which is necessary for the activation of CDKs that will drive the G1–S phase progression^105^. In the absence of androgen signaling, prostate cancer cells will arrest in early G1 phase as they do not have expression of the necessary CDK and cyclin proteins^106,107^. AR and pRb have also been shown to interact, and an overexpression of pRb increases the transcriptional activity of AR^108,109^.

AR signaling is also important for the regulation of other cell cycle related genes, including the regulation of *CCND1* expression^110^. Importantly, *CCND1* encodes cyclin D1 which has an interaction with pRb that is necessary for cell cycle progression. AR binds to AREs that are located ~570–556 base pairs upstream of the transcription start site of the proximal promoter of *CCND1*, suggesting that AR plays a regulatory role to influence *CCND1* expression^110^. In prostate cancer cells, following treatment with androgens, there is induction of expression of CDK inhibitors p21 and p27^111^. Expression of p21 is controlled at the transcriptional level through the presence of an ARE in the promoter region, ~200 base pairs upstream of the proximal promoter^112^. AR signaling has been shown to be important for control of cell cycle-related gene expression, resulting in growth implications in tumor cells.

Additionally, in prostate cancer cells, the synthetic androgen mibolerone inhibited proliferation and reduced levels of c-MYC transcripts, suggesting that AR is important for regulating c-MYC levels^113^. AR expression is also regulated by AREs as well as MYC binding at the consensus site^114^. Thus, in addition to its role cell growth and the DNA damage response, AR expression and activation is itself regulated in a cell cycle-dependent manner which then influences expression of CDK and transcription factors to regulate progression through the cell cycle.

### Breast cancer

In addition to interactions with cyclins and CDKs, AR also interacts with many other important proteins, including well characterized oncogenes and tumor suppressers. In AR+ TNBC, DHT has been shown to increase levels of cyclin D1, while decreasing p73 and p21 expression^115^. Conversely, treatment with bicalutamide resulted in a decrease in cyclin D1 expression, while increasing p73 and p21 levels, implicating a role for AR in the control of cell cycle progression in AR+ TNBC models^115^. The expression of AR and pRb in breast cancer is also significantly correlated, and AR has been shown to interact with other transcription factors, including MYC, which are important for cell cycle control^116^. In breast tumors, high AR expression is negatively correlated with MYC overexpression^116^. MYC expression has been linked to cell proliferation, and inactivation of MYC impairs cell cycle progression as MYC targets cell cycle regulators like cyclins, CDKs, and E2F transcription factors^117^. Additionally, in breast cancer models, the presence of an ARE −383 to −377 base pairs upstream of the ERβ promoter region results in enhanced control of ERβ expression as a result of AR signaling^33^. ERβ has been shown to negatively regulate transcription of c-MYC, cyclin D1, and cyclin A, while also increasing transcription of CDK inhibitors like p21 and p27^33^. In ER+ breast cancer models, DHT-mediated activation of AR has been shown to inhibit ERα signaling and cell cycle progression through a reduction in cyclin D1 transcription^118^. Further, AR and ER both require the steroid receptor coactivator AIB1^118^ which is commonly expressed in breast cancers^119,120^, and high AIB1 expression is correlated with poor mortality^121^. Therefore, through direct or indirect mechanisms, AR signaling likely also plays an important role in controlling cell cycle progression in breast cancer.

## Metastasis

### Prostate cancer

AR has been shown to contribute to the formation of metastases. The AR pathway and AR splice variants have been implicated in metastatic phenotypes in prostate cancer^122^. Gene array and IHC data of both primary and metastatic tumors demonstrate that AR mRNA and protein expression are significantly higher in metastases compared to primary prostate lesions^123^. In vitro, increased AR expression in prostate tumors also led to the formation of metastases and induction of the epithelial to mesenchymal transition (EMT)^123^, the process by which cells lose their polarity and gain the ability to migrate and become invasive. In addition, during prostate cancer development, the presence of fibroblasts provides important structural and functional changes that regulate the extracellular matrix^124^. Expression of nuclear receptors has been shown to be important in squamous cell carcinoma cancer-associated fibroblasts (CAFs) compared to normal-associated fibroblasts, with nuclear receptors influencing many cellular functions including invasiveness^125^. Additionally, AR expression in prostate CAFs has been shown to promote growth and invasion^126^. AR activation in the stroma has been shown to be essential for prostate cancer progression and metastasis^127^. Interestingly, the AR cistrome in prostate CAFs is distinct from the AR cistrome in epithelial cells suggesting a novel role for AR in the microenvironment^128^. Notably, AR relies on AP-1 in the stroma, rather than FOXA1 as observed in epithelial cells^128^.

Furthermore, the regulatory role of AR in gene expression has been shown to be important for the regulation of prostate cancer metastases. In this context, AR negatively regulates expression of ZBTB46, a tumor promoter through miR-1^129^. Therefore, disruption of AR signaling can result in overexpression of ZBTB46 resulting in an increase in transcriptional regulation of SNAI1, a driver of EMT, resulting in metastasis formation^129^. Further, AR inhibition with enzalutamide has been shown to increase metastases by decreasing EPHB6 suppression leading to JNK signaling resulting in cell invasion^130^. These findings suggest that AR plays an important role in controlling metastatic progression of prostate tumors, demonstrating the importance of future work in this area.

### Breast cancer

In patients with breast cancer, metastases are likely to have multiple drivers of disease progression. In preclinical models, AR has also been shown to contribute to invasiveness and migration of TNBC cells through activation of the Src complex^131^. When MCF-7 cells were treated with DHT, there was also an increase in invasion and migration, as well as a decrease in epithelial markers and an increase in mesenchymal markers^132^. DHT treatment also induced other markers of EMT suggesting that AR activation may promote EMT in MCF-7 cells^132^.

As with prostate cancer, previous data from breast cancer patients demonstrates that AR expression is conserved from the primary tumor into metastases^133,134^. One study suggests that there is 78.6% agreement in AR status in primary tumor and lymph node metastases^135^. In the discordant cases, 60/72 had AR- primary tumors, and AR+ lymph node metastases^135^. Further, IHC analyses in tumors and metastases showed greater than 60% agreement between the expression of AR in primary tumor and metastases^136^.

## Treatments targeting androgens and the AR for prostate and breast cancer

**Fig. 1:**
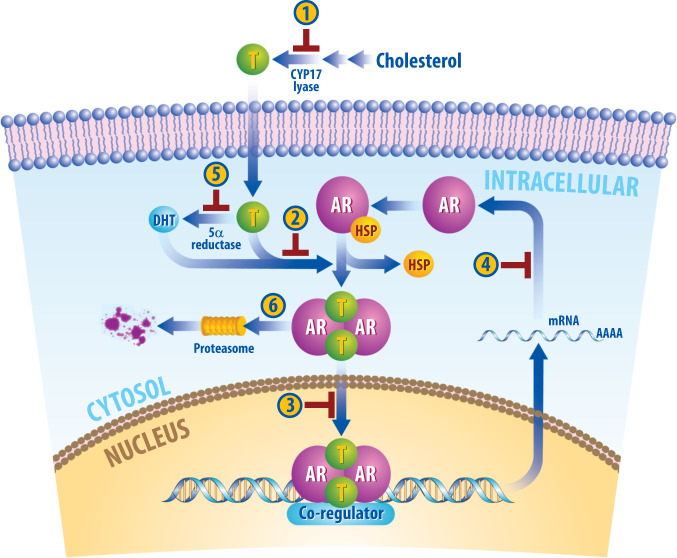
Therapeutic strategies used to inhibit AR signaling. Androgens like testosterone (T) are produced from cholesterol. CYP17-lyase inhibitors and aromatase inhibitors (1), including abiraterone acetate, seviteronel, CR1447, orteronel, galeterone, block the conversion of cholesterol into testosterone. Additionally, luteinizing hormone-releasing hormone (LHRH) or gonadotrophin-releasing hormone (GnRH) antagonists function to reduce levels of circulating androgens, and ketoconazole inhibits production of testosterone. When androgens enter the cell, they can be converted to DHT, a more potent AR agonist. This reaction requires 5α-reductase, an enzyme that can be inhibited by finasteride and dutasteride (5). Antiandrogens (2), including flutamide, bicalutamide, enzalutamide, apalutamide, darolutamide, CR1447, seviteronel, and TRC253, block androgen binding to AR or inhibit AR function. Many antiandrogens, including enzalutamide, apalutamide and darolutamide, inhibit nuclear translation of AR, preventing DNA-binding and downstream gene transcription. Antisense oligonucleotides bind to mRNA encoding AR, preventing protein translation (4). AR degraders (6) promote protein ubiquitination and proteasomal-mediated degradation to lower AR protein levels intracellularly.

Pharmacological agents have been developed to inhibit AR binding to androgens and AR activation due to its role in driving cancer development and progression (Fig. 1). Many of these agents have been effective in the treatment of prostate cancer, and the clinical applications have been expanded to women with AR+ breast cancers. Here we explore these various agents, their mechanisms of action, and the data that exist in the treatment with women with breast cancer, including the ongoing clinical trials assessing their use in women with AR-positive breast cancer and the emerging results from these trials (Tables 1 and 2).

**Table 1:** Current clinical trials in women with breast cancer assessing the safety and/or efficacy of androgen receptor inhibition.

NCT number	Title	Category	AR agent	Additional interventions	Phase
NCT03444025	Neoadjuvant goserelin for triple-negative breast cancer	ADT	Goserelin	Chemotherapy	Phase 2
NCT01352091	Adjuvant AI combined with Zoladex	ADT	Goserelin	Anastrozole, tamoxifen	Phase 3
NCT03878524	A personalized medicine study for patients with advanced cancer of the breast, prostate, pancreas or those with refractory acute myelogenous leukemia	CYP17-lyase inhibitor/antiandrogen	Abiraterone/Enzalutamide	Abiraterone, enzalutamide, venetoclax, palbociclib, all-trans retinoic acid, bortezomib, cabazitaxel, oxaliplatin, fluorouracil, folinic acid, carboplatin, panobinostat, vorinostat, pembrolizumab, bevacizumab, ipilimumab, nivolumab, everolimus, sirolimus, celecoxib, olaparib, afatinib, cabozantinib, sorafenib, dasatinib, erlotinib, idelalisib, imatinib, lenvatinib, pertuzumab, ponatinib, ruxolitinib, sunitinib, trametinib, vemurafenib	Phase 1
NCT03090165	Ribociclib and bicalutamide in AR+ TNBC	Antiandrogen	Bicalutamide	Ribociclib	Phase 1/2
NCT02353988	AR-inhibitor bicalutamide in treating patients with TNBC	Antiandrogen	Bicalutamide	Physician’s choice	Phase 2
NCT03650894	Nivolumab, Ipilimumab, and bicalutamide in human epidermal growth factor (HER) 2 negative breast cancer patients	Antiandrogen	Bicalutamide	Nivolumab, ipilimumab	Phase 2
NCT02299999	SAFIR02_Breast—Efficacy of genome analysis as a therapeutic decision tool for patients with metastatic breast cancer	Antiandrogen	Bicalutamide	Targeted therapies, chemotherapy	Phase 2
NCT03055312	Bicalutamide in treatment of androgen receptor (AR) positive metastatic triple-negative breast cancer	Antiandrogen	Bicalutamide	TPC chemotherapy	Phase 3
NCT03383679	Study on androgen receptor and triple-negative breast cancer	Antiandrogen	Darolutamide	Capecitabine	Phase 2
NCT03207529	Alpelisib and enzalutamide in treating patients with androgen receptor and PTEN positive metastatic breast cancer	Antiandrogen	Enzalutamide	Alpelisib	Phase 1
NCT02689427	Enzalutamide and paclitaxel before surgery in treating patients with stage I–III androgen receptor-positive triple-negative breast cancer	Antiandrogen	Enzalutamide	Paclitaxel, surgery	Phase 2
NCT02953860	Fulvestrant plus enzalutamide in ER+/Her2− advanced breast cancer	Antiandrogen	Enzalutamide	Fulvestrant	Phase 2
NCT02955394	Preoperative fulvestrant with or without enzalutamide in ER+/Her2− breast cancer	Antiandrogen	Enzalutamide	Fulvestrant	Phase 2
NCT02676986	Short-term preoperative treatment with enzalutamide, alone or in combination with exemestane in primary breast cancer	Antiandrogen	Enzalutamide	Exemestane	Phase 2
NCT00755885	Abiraterone acetate in treating postmenopausal women with advanced or metastatic breast cancer	CYP17-lyase inhibitor	Abiraterone acetate		Phase 1/2
NCT01990209	Orteronel as monotherapy in patients with metastatic breast cancer (MBC) that expresses the androgen receptor (AR)	CYP17-lyase inhibitor	Orteronel		Phase 2
NCT01616758	Phase II study of GTx024 in women with metastatic breast cancer	SARM	Enobosarm		Phase 2
NCT02463032	Efficacy and safety of GTx-024 in patients with ER+/AR+ breast cancer	SARM	Enobosarm		Phase 2
NCT02144051	Phase I open label dose escalation study to investigate the safety and pharmacokinetics of AZD5312 in patients with androgen receptor tumors	Antisense oligonucleotides	AZD5312		Phase 1

**Table 2:** Results from completed and ongoing clinical trials investigating the use of androgen receptor inhibition in women with breast cancer.

NCT number	Title	Phase	Treatments tested	Actual or planned patients	Primary endpoint	Secondary endpoints	Three most common adverse events
NCT00186121	Estradiol suppression for the treatment of metastatic breast cancer in premenopausal women	Phase II single arm	Anastrazole + Goserelin	35 pts	ORR: 37.5% (95% CI: 21–56%)	CBR: 71.9% (95% CI: 53–86%) Response rate: CR: 1 pt (3%), PR: 11 pts (34%), SD: 11 pts (34%) TTP: 8.3 (2.1 to NA)^c^ OS: NA (11.1 to NA)^d^ SAE: 0 Estradiol suppression at baseline: 7.47 pg/mL; 1 month: 20.8 pg/mL; 3 months: 18.7 pg/mL; 6 months: 14.8 pg/mL	Hot flush (60%) Arthralgia (53%) Fatigue (50%)
NCT02067741	CR1447 in endocrine responsive-HER2neg and AR+ TNBC	Phase I/II	CR1447	29 pts	MTD: 400 mg/day	DC at 24 weeks: 0 pts (0%) SD at 12 weeks: 2 pts (14%) PD at 12 weeks: 11 pts (79%) 4-OHT *T*_max_: 16 h (range: 1.0–72.0) 4-OHT *C*_*max*_: 0.63 ng/mL (range: 0.0–1.88) median AUC0-72: 27.2 h ng/mL (range: 0.0–69.8)	Elevated triglycerides (57%) Anemia (50%) Elevated AST (29%) Elevated AP (29%) High creatinine (29%)
NCT00468715	Bicalutamide in treating patients with metastatic breast cancer	Phase II	Bicalutamide	28 pts	CBR (6 months): 19% (95% CI: 7–39%) CBR (6 months, ITT): 18% (95% CI: 6–37%)	Median PFS: 12 weeks (95% CI: 11–22 weeks)	Elevated AST (25%) Fatigue (21%) Hot flashes (21%) Limb edema (21%)
NCT02910050	Bicalutamide plus aromatase inhibitors in ER(+)/AR(+)/HER2(−) metastatic breast cancer	Phase II single arm	Bicalutamide + Aromatase inhibitors	58 pts	CBR (6 months): 16.7% CR: 0 pts (0%) PR: 0 pts (0%) SD: 3 pts (17%) PD: 15 pts (83%)	PFS: 2.7 months (95% CI: 2.2–3.8 months)	Tumor pain (17%) Alopecia (6%) Hot flashes (6%) Peripheral sensory neuropathy (6%) Insomnia (6%) Hypertension (6%)
NCT02605486	Palbociclib in combination with bicalutamide for the treatment of AR(+) metastatic breast cancer (MBC)	Phase I/II	Bicalutamide + Palbociclib	51 pts	The MTD was 150 mg bicalutamide daily and 125 mg palbociclib daily for 21 days in a 28 day cycle.		Neutropenia (33%) Leukopenia (27%) Lymphocytopenia (20%)
NCT02457910	Taselisib and enzalutamide in treating patients with androgen receptor positive triple-negative metastatic breast cancer	Phase I/II	Enzalutamide + Taselisib	73 pts	MTD was not reached: 160 mg enzalutamide with 4 mg taselisib had manageable toxicities. CBR (16 weeks, evaluable population): 35.7%	PFS (evaluable population): 3.4 months	Phase I: metabolism and nutrition (25%), rash maculopapular (25%), rash acneiform (8%), elevated alkaline phosphatase (8%) Phase II: rash maculopapular (29%), rash acneiform (12%), fatigue (12%)
NCT01597193	Safety study of enzalutamide (MDV3100) in patients with incurable breast cancer	Phase I	Enzalutamide ± Aromatase inhibitors/SERD	101 pts	MTD not yet reported. 160 mg enzalutamide: 22 patients, 3 AE 160 mg enzalutamide + 1 mg anastrozole: 20 patients, 1 AE 160 mg enzalutamide + 50 mg exemestane: 23 patients, 3 AEs 160 mg enzalutamide + 500 mg fulvestrant: 11 patients, 2 AEs	Enzalutamide: 4 pts with ≥ Grade 3 AE; 1 pt discontinued treatment due to AEs Enzlautamide + Anastrozole: 6 pts with ≥ Grade 3 AE; 1 pt discontinued treatment due to AEs Enzalutamide + Exemestane: 9 pts with ≥ Grade 3 AE; 3 pts discontinued treatment due to AEs Enzalutamide + Fulvestrant: 4 pts with ≥ Grade 3 AE Maximum plasma concentration (Cmax) of enzalutamide and metabolites after single dosing (enzalutamide 160 mg) [μg/mL]: enzalutamide: 4.01 (2.09); M1 (carboxylic acid): 0.0707 (0.0379); M2 (N-desmethyl): 0.184 (0.0689) AUC 24 h after single dosing (enzalutamide 160 mg) [μg h/mL]: enzalutamide: 41.6 (8.19); M1: 1.20 (0.648); M2: 2.76 (1.00) Terminal elimination half life after single dosing (Enzalutamide 160 mg): 198 h (105)	Enzalutamide: nausea (50%), fatigue (45%), back pain (27%), cough (27%) Enzalutamide + Anastrozole: fatigue (60%), decreased appetite (50%), nausea (45%) Enzalutamide + Exemestane: fatigue (52%), nausea (52%), vomiting (30%) Enzalutamide + Fulvestrant: fatigue (73%), nausea (73%),back pain (55%)
NCT02091960	A study to assess the efficacy and safety of enzalutamide with trastuzumab in patients with human epidermal growth factor receptor 2 positive (HER2+), androgen receptor positive (AR+) metastatic or locally advanced breast cancer	Phase II single arm	Enzalutamide + Trastuzumab	103 pts	CBR: 23.6% (95% CI: 15.2–33.8%)	ORR at week 24: 3.4% (95% CI: 0.7–9.5) Best ORR: 4.5% (95% 1.2–11.1) PFS: 105 days (95% CI: 61–116) TTP: 108 days (95% CI: 61–116) Duration of response: NA^e^ Time to response: 57 days (95% CI: 57–222) Patients with AEs: 94% (related to enzalutamide 73%, related to trastuzumab 38%)	Fatigue (34%) Nausea (27%) Hot flush (17%)
NCT02007512	Efficacy and safety study of enzalutamide in combination with exemestane in patients with advanced breast cancer	Phase II	Enzalutamide + Exemestane vs. Placebo + Exemestane	247 pts	Enzalutamide + Exemestane: PFS (ITT): 11.8 months (7.3–15.9); PFS (DX+): 16.5 months (11.0-NA^a^) Enzalutamide: PFS (ITT): 5.8 months (3.5–10.9); PFS (DX+): 4.3 months (1.9–10.9) HT + Enzalutamide + Exemestane: PFS (ITT): 3.6 months (1.9–5.5); PFS (DX+): 6.0 months (2.3–26.7) HT + Enzalutamide: PFS (ITT): 3.9 months (2.6–5.4); PFS (DX+): 5.3 months (1.8–6.7)	Enzalutamide + Exemestane: CBR 24 weeks: 62% (49–74%); best objective response rate: 31% (17–48%); duration of objective response: 14.0 months (5.6-NA^a^); time to response: 12.9 months (7.3-NA^a^); time to progression: 11.8 months (7.3–15.9); PFS at 6 months: 67% (53–77%) Enzalutamide: CBR 24 weeks: 45% (33–58%); best objective response rate: 19% (9–34%); duration of objective response: 9.1 months (3.2–10.2^a^); time to response: 14.0 months (7.4-NA^a^); time to progression: 7.4 months (3.5–13.5); PFS at 6 months: 50% (37–62%) HT + Enzalutamide + Exemestane: CBR 24 weeks: 20% (11–32%); best objective response rate: 10% (3–23%); duration of objective response: 18.3 months (3.3–23.1); time to response: NA (3.9-NA^a^); time to progression: 3.6 months (1.9–5.6); PFS at 6 months: 32% (20–44%) HT + Enzalutamide: CBR 24 weeks: 32% (20–45%); best objective response rate: 5% (0.6–16%); duration of objective response: 4.6 months (1.9–7.4); time to response: NA (NA-NA^a^); time to progression: 3.9 months (2.6–5.4); PFS at 6 months: 33% (22–46%)	Combined from all arms: fatigue (32%), nausea (26%), hot flush (23%)
NCT01889238	Safety and efficacy study of enzalutamide in patients with advanced, androgen receptor-positive, triple-negative breast cancer	Phase II single arm	Enzalutamide	118 pts	CBR (16 weeks, evaluable population): 33% (95% CI: 26–42%) CBR (16 weeks, ITT): 25% (95% CI: 19–31%)	CBR (24 weeks, evaluable population): 28% (95% CI: 21–36%) CBR (24 weeks, ITT): 20% (95% CI: 15–26%) Best objective response (evaluable population): 8.5% (95% CI: 3–12%) Best objective response (ITT): 6% (95% CI: 3–9%) PFS (evaluable population): 14.3 weeks (95% CI: 8.3–16.1) PFS (ITT): 12.6 weeks (95% CI: 8.1–15.1)	Fatigue (42%) Nausea (34%) Decreased appetite (19%)
NCT02750358	Feasibility study of adjuvant enzalutamide for the treatment of early stage AR (+) triple-negative breast cancer	Phase III	Enzalutamide	50 pts	As of 6/27/19, 34 pts (68%) completed 1 year of treatment, and 15 pts (30%) were off treatment.		Fatigue (48%) Hot flashes (22%) Headache (18%) Hyperglycemia (18%) Nausea (18%)
NCT03004534	A study to evaluate changes in human breast cancer tissue following short-term use of darolutamide	Early Phase I single arm	Darolutamide	36 pts	Presurgical molecular assessment: AR up (7 pts, 20.6%) AR unchanged (12 pts, 35.3%) AR down (15 pts, 44.1%)	26 pts with TEAE (72%) 10 pts with no TEAE (28%)	Fatigue (22%) Constipation (8%) Diarrhea (8%) Nausea (8%)
NCT02580448	CYP17-lyase and androgen receptor inhibitor treatment with seviteronel trial (CLARITY-01)	Phase I/II	Seviteronel	175 pts	CBR (16 weeks, TNBC): 2 pts (33%)^b^ CBR (24 weeks, ER + BC): 2 pts (18%)^b^	Change in CTC at C2D1: −94.3% (range: −27.5,−100)^b^	Fatigue (50%) Nausea (43%) Decreased appetite (33%)
NCT01842321	Abiraterone acetate in molecular apocrine breast cancer	Phase II single arm	Abiraterone acetate + Prednisone	31 pts	CBR (6 months): 20.0% (95% CI: 8–39%). CR (6 months): 1 pt (3%) PR (6 months): 0 pt (0%) SD (6 months): 5 pts (17%) Progression at 6 months: (23 pts (77%) Treatment stopped for toxicity before 6 months evaluation: 1 pt (3%)	ORR: 6.7% (95% CI 0.8–22%) DoR: CR: 23.4 months; PR: 5.6 months PFS: 2.8 months (95% CI: 1.7–5.4)	Fatigue (18%) Hypertension (12%) Hypokalemia (9%)
NCT00212095	Docetaxel combined with ketoconazole in treatment of breast cancer	Phase II	Ketaconazole + Docetaxel	30 pts	Cycles of docetaxel: 4 (ketoconazole); 6 (conventional) Ketoconazole-dosed Docetaxel: 52% of pts had reduction in tumor dimension; CR: 9.7%; PR: 54.8%; ORR: 64.5; SD: 4.1%; PD: 77.6% Conventional-dosed docetaxel (doxirubicin): 55% of pts had reduction in tumor dimension; CR: 4.1%; PR: 776%; ORR: 81.7%; SD: 16.3%; PD: 2.0%	AUC (mg/L h): ketaconazole-modulated docetaxel: 3.93 ± 2.77; conventional-dosed docetaxel: 3.77 ± 2.70 [*p*-value = 0.794] Clearance (L/h): ketaconazole-modulated docetaxel: 22.05 ± 8.29; conventional-dosed docetaxel: 36.52 ± 13.39 [*p*-value < 0.001] Half-life (h): ketaconazole-modulated docetaxel: 13.46 ± 5.05; conventional-based docetaxel: 12.25 ± 3.47 [*p*-value = 0.206] *C*_*max*_ (mg/L): ketaconazole-modulated docetaxel: 2.53 ± 1.14; conventional-based docetaxel: 2.68 ± 1.09 [*p*-value = 0.568]	Fatigue (81%) Diarrhea (58%) Myalgia (36%)
NCT01808040	A Phase 1b study of TAK-700 in postmenopausal women with hormone-receptor positive metastatic breast cancer	Phase Ib	Orteronel	8 pts	MTD not yet reported. Dose level 1: 300 mg (4 pts, 1 not evaluated) Dose level 2: 400 mg (3 pts)	1 patient with SD > 6 months 1 patient with SD for 3 months	Hot flashes (28%) Nausea (28%) Hypokalemia (28%) Elevated AST (28%)
NCT02971761	Pembrolizumab and enobosarm in treating patients with androgen receptor positive metastatic triple-negative breast cancer	Phase II	Enobosarm + Pembrolizumab	29 pts	PR: 2 pts (13%) SD at 18 and 19 weeks: 2 pts (13%) PD: 11 pts (69%)		Elevated liver function (19%) Diarrhea (13%) 6% of the following: adrenal insufficiency, dry skin, headache, hot flashes, hyperhidrosis, hyperthyroidism, or palpitation

*AE* adverse events, *CBR* clinical benefit rate, *CI* confidence interval, *CR* complete response, *CTC* circulating tumor cells, *DC* disease control, *DLT* dose-limiting toxicities, *DoR* duration of overall response, *DX*+ diagnostic positive, *HT* prior hormone therapy treatment, *ITT* intent to treat, *MTD* maximum tolerated dose, *ORR* objective response rate, *PD* progressive disease, *PFS* progression-free survival, *PR* partial response, *SAE* serious adverse events, *SD* stable disease, *TEAE* treatment-related adverse events, *TTP* time to progression.

^a^Upper limit of 95% confidence interval or median was not reached due to insufficient number of events at the time of data cut-off.

^b^Only Phase 2 Stage 1 results have been reported.

^c^Upper limit of TTP range was not determined/reached.

^d^The median and upper limit of the range for OS were not reached/not determined. The upper limit exceeded 63 months.

^e^Could not be estimated due to low number of events.

### Androgen deprivation therapy

The use of ADT is universally accepted as a first line therapy for metastatic prostate cancer^137^. This treatment attempts to lower levels of serum testosterone in men with prostate cancer to prevent tumor growth^138^. This is done chemically with the use of luteinizing hormone-releasing hormone (LHRH) or gonadotrophin-releasing hormone (GnRH) antagonists, like Degarelix, Goserelin, and Leuprolide, which are used to suppress the production of androgens^137^. Many clinical trials also are assessing the efficacy of ADT in combination with other treatment strategies in an attempt to improve ADT efficacy, especially in cases where AR mutations cause castration to be ineffective at controlling disease progression.

### 5α-reductase inhibitors

5α-DHT is produced from testosterone in specific tissues, including the prostate, through the enzymatic activity of 5α-reductase. Compared to testosterone, DHT has a slower dissociation rate from AR, suggesting that AR-DHT is a more stable complex, making DHT the preferred AR ligand^139,140^. Competitive inhibitors of 5α-reductase, like finasteride or dutasteride, can be used to lower levels of serum and prostate DHT^141–144^. The effects of these 5α-reductase inhibitors, however, are complex as they may not exclusively target the enzymatic activity of 5α-reductase and likely have additional off-target AR inhibitory effects as well^145^.

### CYP17-lyase inhibitors

Abiraterone acetate is a selective inhibitor of cytochrome P450 17alpha-hydroxylase/17,20-lyase (CYP17), which, through its function, decreases the adrenal and tumoral synthesis of androgens^146^. CYP17-lyase inhibitors lower androgen availability to reduce the activation of androgen signaling. Trials in men with chemotherapy-naive CRPC concluded that treatment with abiraterone acetate and prednisone prolongs overall survival compared to treatment with prednisone alone (NCT00887198)^147^. In a phase II trial for women with triple negative, AR+ locally advanced or metastatic breast cancer (NCT01842321), treatment with abiraterone acetate and prednisone also provided benefit for some patients^148^. Of 138 patients assessed for the trial, 53 (37.6%) had AR+ TNBC: 34 of these patients were included. This trial assessed the clinical benefit rate (CBR) for 30 of the patients at 6 months with a CBR of 20.0% (95% CI: 7.7–38.6%) including one patient who had a complete response (CR) and 5 patients with stable disease (SD) at ≥6 months^148^. Secondary outcomes included objective response rate (6.7%, 95% CI: 0.8–22.1%), and progression-free survival (median time: 2.8 months, 95% CI: 1.7–5.4)^148^. These studies suggest that treatment with abiraterone acetate may be a beneficial treatment strategy for both men with CRPC and women with molecular apocrine breast cancer.

Other CYP17-lyase inhibitors include galeterone (TOK-001) and orteronel (TAK-700)^149^. Galeterone has been shown to be effective in reduction of PSA levels and was well-tolerated by patients in early clinical trials^150^. Orteronel treatment is effective at suppressing testosterone levels and shrinking the androgen-dependent organs including the prostate gland^151^. Phase III clinical trials found that orteronel and prednisone treatment verses placebo and prednisone gave patients longer progression-free survival (PFS); however, men with orteronel and prednisone treatment did not have extended overall survival^152^. In breast cancer, there are currently phase I and II clinical trials assessing the use of orteronel in patients with metastatic breast cancers that express AR (NCT01808040, NCT01990209). NCT01808040 assesses the safety of orteronel use for the treatment of postmenopausal women with hormone receptor positive metastatic breast cancer in addition to measuring the estradiol levels in these patients following treatment^153^. NCT01990209 is a phase II trial for male or female patients with metastatic AR+ BC (TNBC or ER+ and/or PR+ BC) with primary outcome measures of response and disease control rates. This trial will also assess safety, PFS, OS, and serum hormone levels in addition to screening tumors for PTEN expression and PIK3CA mutations. Due to failure in phase III clinical trials in men with prostate cancer, orteronel was taken out of development in 2014^154^.

### Antiandrogens

Antiandrogens are a class of agents which act as nonsteroidal competitive inhibitors of the AR^155^. Flutamide and bicalutamide are two such agents that have been used to block androgen binding and abrogate nuclear AR signaling. Although AR targeting has been a strategy for over 30 years, original phase II clinical trials with flutamide suggested it did not have antitumor activity which delayed the initiation of further trials with the drug^156^. Recent studies, however, have shown that flutamide treatment is effective and well-tolerated for treating PSA recurrence following prostatectomy, radiation therapy, or cryotherapy for patients with prostate cancer^157^. In addition, in breast cancer, bicalutamide has been shown to have a CBR of 19% in patients with AR+, ER−, PR− metastatic breast cancer where 12% of tumors were AR+^158^. These results suggest that antiandrogen therapies are effective for the treatment of patients with traditionally hormone receptor-negative breast cancers. Unfortunately, in prostate cancer, it has been shown that exposure to antiandrogens can augment frequency of AR mutations and variants^159^, and metabolites of antiandrogens can result in stimulation of prostate cancer cell growth as flutamide metabolites function as an AR agonist^160^. There are additional ongoing clinical trials that are assessing the use of flutamide as a second line treatment of patients with CRPC who have relapsed after ADT and bicalutamide treatment (NCT02918968) or using flutamide treatment to prevent prostate cancer in patients with neoplasia of the prostate (NCT00006214). In addition, NCT02910050 is investigating the use of bicalutamide with AIs in AR+, ER+ breast cancers^161^.

### Second generation antiandrogens

Four FDA-approved second generation antiandrogens, abiraterone acetate, apalutamide, darolutamide, and enzalutamide, improve upon the first-generation antiandrogens. Enzalutamide is able to inhibit the growth of both ER+ and ER− breast tumors by inhibiting AR nuclear translocation^146^. In addition to growth inhibition, enzalutamide also can inhibit tumor cell migration and invasion^162^. In mCRPC patients who had previously received chemotherapy treatment, treatment with enzalutamide also contributed to prolonged survival (NCT00974311)^163^. In breast cancer, a Phase II trial (NCT01889238) for women with advanced, AR+ TNBC tested the use of enzalutamide for improving outcomes and CBR for patients at 16 weeks (CBR16) as well as assessing clinical benefit at 24 weeks (CBR24), PFS, response rates, and safety of enzalutamide treatment^164^. This study also found that 47% of the 118 enrolled patients had an AR related gene signature, and clinical outcomes were better for patients with AR+ disease^164^. No new side effects were reported from enzalutamide treatment in this trial, indicating its potential use as a therapeutic for women with TNBC^164^.

Apalutamide (ARN-509) is a second generation AR antagonist, similar to enzalutamide, that binds to the LBD of AR to inhibit nuclear translocation and ARE binding^149^. Apalutamide has a seven- to ten-fold increased binding affinity to AR compared to bicalutamide^165^. In preclinical studies, apalutamide had antitumor activity in a castration-sensitive model of prostate cancer^166^. There were also lower levels of apalutamide in mouse steady-state plasma and brain levels compared to enzalutamide treatment, which could indicate lower frequency of seizures with apalutamide^166^. In preclinical studies, apalutamide also had antitumor and growth inhibitory effects in AR+ TNBC cells^166^. Results from the SPARTAN trial, a Phase III clinical trial (NCT01946204) for men with nonmetastatic castration-resistant prostate cancer (nmCRPC), demonstrated improved metastasis-free survival in patients treated with apalutamide compared to placebo^167^. Following this trial, apalutamide was approved by the FDA for treatment of nmCRPC^165^. To date, there have been no trials with apalutamide in patients with AR+ breast cancer.

Darolutamide (ODM-201) is an AR inhibitor that binds wild-type AR with a higher affinity than enzalutamide to block AR nuclear translocation^149^. In addition, darolutamide can be effective against mutant ARVs which can develop with resistance to enzalutamide and apalutamide therapy^168^. In prostate models, darolutamide has low brain-penetrance and treatment does not produce an increase in mouse serum testosterone levels^168^. Recently, results from the ARAMIS trial (NCT02200614), a phase III trial for nmCRPC patients, demonstrate that darolutamide provides better metastasis-free survival compared to placebo^169^. The START trial is a phase II trial for women with AR+ TNBC comparing darolutamide treatment with capecitabine, an antimetabolite chemotherapeutic (NCT03383679). This trial investigates CBR16 as a primary objective, and CBR24, response rates, overall survival, PFS, and safety as secondary objectives for women with locally recurrent or metastatic AR+ TNBC.

### Novel compounds

A number of novel compounds have also been developed to block or abrogate androgen signaling. Seviteronel (VT-464) is a nonsteroidal selective CYP17-lyase inhibitor and AR antagonist that both blocks testosterone and estrogen production and inhibits AR activation^170^, rendering it a potentially effective alternative to agents which either inhibit androgen production or AR activation. Clinical trials for patients with ER+ or TNBC indicated that seviteronel was well-tolerated in women, with the majority of adverse events (AEs) being Grade 1/2, in addition to four Grade 3/4 AEs that may be related to seviteronel treatment^171^. Phase I trials in CRPC patients suggest that seviteronel may be an effective treatment alternative for men who are not responsive on other therapies with most reported AEs being Grade 1/2^172^. Preclinical work in AR+ TNBC demonstrates that seviteronel inhibits cell proliferation and growth on soft agar^173^. ChIP-seq and RNA-seq analyses demonstrate that AR-regulated genes are increased with DHT stimulation and decreased in mice treated with seviteronel^173^. Trials with seviteronel continue to be ongoing for patients with CRPC, AR+ TNBC, or men with ER+ breast cancer, who had previously been treated with enzalutamide (NCT02130700, NCT02012920, NCT02580448, NCT03600467). The CLARITY-01 trial (NCT02580448) is assessing the CBR at 16 or 24 weeks for women with ER+ or TNBC or men with locally advanced or metastatic breast cancer who are receiving seviteronel treatment^174^. Of the patients enrolled for stage 1, CBR16 for TNBC patients was 2 of 6, and CBR24 for ER+ patients was 2 of 11^174^. Of patients with CTCs, 7 of 10 had a CTC decline at C2D1^174^. Patients receiving seviteronel also had a decline from baseline in concentrations of estradiol and testosterone^175^. The most common AEs were tremor, pain, fatigue and dyspnea, nausea, AST increase, ALT increase and abdominal pain, suggesting that seviteronel was well-tolerated^175^. These results indicate that seviteronel may be a potential therapeutic option for the treatment of AR+ disease.

CR1447 (4-hydroxytestosterone [4-OHT]) is a novel AR inhibitor that acts both as a steroidal AI as well as an AR antagonist by binding to AR^176^. When injected, 4-OHT is converted to 4-hydroxyandrostenedione (4-OHA), a previously used form of AI that was injected for the treatment of breast cancer^176^. Both 4-OHT and 4-OHA are unable to be made into estrogens in vivo^176^. Preclinically, CR1447 has been shown to inhibit growth of AR+ BC cell lines, but not AR knockout cell lines, or those with siRNA-mediated AR knockdown^176^. Results from a Phase I clinical trial (NCT02067741) indicate that, when topically administered, CR1447 was well-tolerated with grade 1/2 AEs and no dose-limiting toxicities (DLTs) in 12 patients with ER+/HER2− breast cancer^176^. Two patients (17%) had stable disease after 12 weeks of treatment^176^. Therefore, CR1447 may also be viable treatment option.

Enobosarm is a selective androgen receptor modulator (SARM) that was originally tested in Phase I, II, and III clinical trials for its use in improving lean body mass and treating cachexia^177^. Enobosarm has tissue specific activity, with anabolic activity in muscles and bone without affecting growth of hair in women and prostate in men^178^. It has been well-tolerated by both men and women; additionally, in patients with advanced cancer, treatment with enobosarm leads to an increase in lean body mass^179^. Enobosarm has also been well-tolerated as an androgen agonist in women with AR+ metastatic breast cancer^180^. Androgen-based AR agonists have previously been shown to be effective for the treatment of breast cancer^181^, and enobosarm similarly stimulates AR, but unlike androgens, does not have masculinizing side effects^182^. A phase II trial (NCT01616758) assesses CBR, and PSA is evaluated as a biomarker of AR activity. In addition, NCT02971761 is investigating the use of pembrolizumab with enobosarm for AR+ TNBC patients^183^. Enobosarm may soon join the treatment armamentarium.

Antisense oligonucleotides (ASOs) have also been used to inhibit AR-driven gene expression, especially in contexts where AR is activated independent of hormone binding. ASOs bind to mRNA, causing the mRNA to be degraded, therefore reducing levels available for protein synthesis. Prostate cancer models have shown that ASOs are able to reduce AR expression, resulting in decreased cell growth^184,185^. In addition, ASOs used against AR mRNA were able to shut down the downstream activation of AR-mediated genes in hormone-independent conditions^186^. ASO administration in mouse models did not have any observed side effects and, compared to castration, did not result in shrinking of mouse prostates^185^. Use of ASOs may also be a method for targeting AR splice variants as two ASOs have been used to effectively silence AR-V7, but not AR-FL, signaling in CRPC cell lines^187^. Therefore, these findings suggest that the use of ASOs may be a useful strategy for overcoming the resistance that often develops to antiandrogens in prostate cancer. In addition, ASOs may also be an effective treatment strategy for targeting mutant ARVs.

Targeted degradation of proteins with the use of Proteolysis Targeting Chimeras (PROTACs) is a novel method for the inhibition of AR signaling in prostate cancer cell models. PROTAC-mediated degradation takes advantage of E3 ubiquitin ligase activity by linking a ligand for the target protein to a ligand for the E3 ubiquitin ligase^188^. Upon ligand binding to the protein of interest, the protein is ubiquitinated by the E3 ubiquitin ligase resulting in degradation by the 26S proteasome. Multiple AR degraders have been developed using PROTAC for use in prostate cancer^189,190^, and they have been shown to be more effective than enzalutamide in vitro and in vivo in models of enzalutamide-sensitive and resistant prostate cancer^191,192^. Enhanced efficacy of AR degraders in prostate cancer models may demonstrate the importance of removing AR protein as opposed to pharmacologically inhibiting AR activity for the treatment of resistant prostate cancers. In the future, pharmacologic AR degraders may be introduced clinically for the treatment of aggressive AR-driven cancers.

There are also additional compounds that have limited use in treating AR-driven disease. Ketoconazole is an antifungal agent that is also able to competitively bind to the AR^193^. Ketoconazole has also been shown to inhibit enzymes important for testosterone synthesis^194^ and is under investigation in combination with docetaxel (NCT00212095)^195^. In addition, TRC253, a novel competitive inhibitor of AR has been shown to be an antagonist to wild-type AR as well as all tested AR mutants^196^, including AR F877L, a mutation occurring in the LBD of AR^197^.

## Combination therapies

### AR + radiation therapy

#### Prostate cancer

Radiotherapy has been shown to induce AR expression in prostate cancer cells, and ADT sensitizes cancer cells to radiotherapy^198^. Treatment with enzalutamide was also shown to radiosensitize prostate cancer cells more effectively than ADT^199^. Combination treatment with enzalutamide and radiation therapy resulted in a significant increase in apoptosis and senescence compared to treatment with enzalutamide or radiation alone^199^. In prostate cancer, radiosensitization was also observed with ARN-509^97^. In addition, treatment with antiandrogen therapies resulted in the downregulation of DNA repair genes, thereby promoting radiosensitivity through a decrease in NHEJ activity^97^.

#### Breast cancer

The AR has been shown to be a potential mediator of radioresistance and a target for the radiosensitization of AR+ TNBC^100–102,200^. Inhibition of AR with enzalutamide results in increased radiosensitization of AR+ breast cancer cells through the inhibition of AR-activated DNA-PKcs-mediated repair^100^. Similar results were observed with seviteronel, the dual CYP17 inhibitor and AR antagonist^101^, however the differences in the mechanisms of radiosensitization with these agents need to be further assessed.

### AR + PARP inhibitors

#### Prostate cancer

Poly ADP-ribose polymerase (PARP) is a nuclear enzyme that modifies substrates through the addition of PAR moieties^201^. Cancers with mutations to *BRCA1* or *BRCA2* have HR deficiencies, rendering them increasingly susceptible to treatment with PARP inhibitors. Inhibition of PARP in tumors with *BRCA* mutations results in synthetic lethality and forces cells to rely on NHEJ for repair of DNA breaks. PARP has been shown to be recruited to sites of AR binding and promotes AR function^201^. When AR is inhibited, HR deficiency and BRCAness is induced^202^. Therefore, AR activity is important for the maintenance of HR gene expression. Following ADT, PARP levels are elevated leading to prostate cancer cell survival^203^. Combination therapy of PARP inhibition with ADT may be important for the impairment of HR before the tumors become castration resistant^203^.

PARP also plays an important role in the AR signaling cascade. Combination treatment with the PARP inhibitor talazoparib with enzalutamide or abiraterone acetate has significant synergy^204^. Antiandrogen therapies induce PARP cleavage, resulting in an increase in dsDNA breaks^204^. This synergy is a therapeutic target for CRPC patients with mutations in DNA damage repair. Therefore, cancer cells with DNA damage repair mutations are more sensitive to PARP inhibitors due to the role of the AR in the transcriptional regulation of DDR genes^205^.

#### Breast cancer

PARPi has been established to be an effective treatment strategy for patients with breast cancers harboring mutant *BRCA1* and *BRCA2*^206^. To date, the combination therapy of PARPi with anti-AR therapy has not been tested in breast cancer; however, this combination may be an effective treatment strategy for AR+ BC patients, especially those with *BRCA* mutated tumors. Because many PARPi can induce PARP trapping, resulting in the formation of dsDNA lesions^207^, and anti-AR therapies have been demonstrated to result in a delay in dsDNA break repair in the presence of DNA damage, combining these therapies may be effective in creating deleterious lesions for tumor cells. Future work may assess PARPi in combination with anti-AR therapies for the treatment of AR+ breast cancers.

### AR + CDK4/6 inhibitors

#### Prostate cancer

AR regulates cell cycle progression through the G1–S phase transition, therefore promoting CDK activity and inducing phosphorylation for the inactivation of pRb^105^. Due to crosstalk of AR with CDK/pRb in promoting cell cycle progression, combined AR and CDK4/6 inhibition has also been shown to be a therapeutic strategy in prostate cancer^208^.

#### Breast cancer

Palbociclib, ribociclib, and abemaciclib are selective inhibitors of CDK4/6 and are widely used for the treatment for ER+ breast cancer. A Phase I/II clinical trial is currently assessing the use of palbociclib with bicalutamide for treatment of AR+ metastatic TNBC (NCT02605486). This trial will establish recommended doses for the combination therapy in addition to measuring PFS, and secondary outcomes including response rates, CBR, and safety^209^.

### AR + PI3K inhibitors

#### Prostate and breast cancer

Phosphatindylinositol 3-kinase (PI3K) is an enzyme involved in cellular functions including cell growth, proliferation, and differentiation; however, PI3K is also highly mutated in cancer. Qi et al. found that inhibition of both AR and PI3K can be synergistic as AR and PI3K signaling work through reciprocal feedback loops^210^. Combined inhibition of AR with the PI3K or mTOR pathway suppressed cell proliferation and resulted in an increase in apoptosis and cell cycle arrest in CRPC cells^210^. An ongoing trial is investigating the treatment of taselisib, a PI3K inhibitor, and enzalutamide in patients with AR+ metastatic TNBC (NCT02457910). This trial will assess dose-limiting toxicities to determine the maximum tolerated dose in addition to measuring patient response and CBR^211^.

#### Phase III development of antiandrogen treatments

Many antiandrogen treatment strategies have been effectively translated from preclinical studies into clinical use through the use of clinical trials. For women with metastatic, AR+ TNBC, there is a phase III clinical trial (NCT03055312) underway comparing conventional chemotherapy to bicalutamide treatment. This trial will assess the CBR at 16 weeks as well as progression-free survival at 24 months. The ENDEAR trial (NCT02929576) is a phase III trial comparing PFS for patients treated with paclitaxel chemotherapy +/− enzalutamide or enzalutamide followed by paclitaxel treatment; however, this trial was withdrawn. Finally, there is an ongoing feasibility trial (NCT02750358) of enzalutamide in women with AR+ TNBC that should report preliminary DFS and OS data in the coming year^212,213^. Data recently presented from this trial reported that enzalutamide treatment is feasible and well-tolerated in this patient population. Finally, phase I/II clinical trials continue to inform drug development and clinical practice, including trials of newer generation antiandrogen agents in women with AR+ breast cancer. Additional studies are needed to better understand use of antiandrogen therapies for the treatment of women with AR+ breast cancers.

## Conclusion

While the role of AR in prostate cancer is more completely understood, the importance of AR signaling in breast cancer is an area of increasing investigation. In order to understand the mechanism of AR signaling and to design proper therapies against AR in breast cancer, additional work needs to be done to elucidate the mechanism by which AR is activating its target genes and contributing to tumor growth and metastasis, as well as systemic and radiation therapy resistance. Advancements in this mechanistic understanding will shed light on potential combination therapies and will allow for more effective treatment for patients with AR+ breast cancers. Further, discerning the intricacies and crosstalk between AR and ER signaling may also provide advancements for treatment of AR+, ER+ breast cancers. These outcomes would be impactful not only for the advanced understanding of the role of AR, but also for new ways in which AR signaling can be inhibited to improve outcomes for women with AR+ breast cancer.
